# BRCA1 founder mutations do not contribute to increased risk of gastric cancer in the Polish population

**DOI:** 10.1186/s13053-015-0043-0

**Published:** 2016-01-15

**Authors:** Małgorzata Ławniczak, Anna Jakubowska, Andrzej Białek, Jan Lubiński, Katarzyna Jaworska–Bieniek, Katarzyna Kaczmarek, Teresa Starzyńska

**Affiliations:** Department of Gastroenterology, Pomeranian Medical University, Unii Lubelskiej 1, Szczecin, 71-252 Poland; Department of Genetics and Pathology, International Hereditary Cancer Center, Pomeranian Medical University, Połabska 4, Szczecin, 70-115 Poland

## Abstract

**Background:**

Gastric cancer (GC) is part of the spectrum of diseases linked to *BRCA1* and *BRCA2* mutations that increase the risk of breast and ovarian cancer. Data suggesting an increased risk of developing GC among *BRCA1* and *BRCA2* mutation carriers are based almost exclusively on indirect studies. The objective was to assess in a direct study whether there is a relationship between GC and selected recurrent *BRCA1* and *BRCA2* mutations in the Polish population.

**Methods:**

Three hundred seventeen GC patients (193 males and 124 females; mean age 59.5 ± 12.8 y) diagnosed at the Department of Gastroenterology at the Pomeranian Medical University were included in this retrospective study. All patients were genotyped for 3 *BRCA1* Polish founder mutations (5382insC, C61G and 4153delA) as well as for 9 known recurrent mutations in *BRCA1* and *BRCA2* genes. Genotyping was performed using allele-specific oligonucleotide polymerase chain reaction (ASA-PCR) for 4153delA and 5382insC, restriction fragment length polymorphism (PCR-RFLP) for C61G and TaqMan real-time PCR for 185delAG, 3819del5, 3875del4, 5370C > T, 886delGT, 4075delGT, 5467insT, 6174delT and 8138del5.

**Results:**

Among tested mutations one founder *BRCA1* mutation 5382insC was detected in two of 317 (0.63 %) GC cases. A comparison of frequency of detected *BRCA1* founder mutations in GC patients to previously described 4570 Polish controls (0.63 % vs. 0.48 %) failed to indicate an increased risk of GC in the mutation carriers (OR = 1.3; 95 % CI 0.3-5.6, *p* = 0.71). A comparison of frequency of GC male cases and male controls (1.0 % vs. 0.43 %,OR = 1.5; 95 % CI 0.3-6.4, *p = 0.61*) allowed to formulate the same conclusion that there is no increased risk for GC for males. None of the 9 recurrent *BRCA1* and *BRCA2* mutations has been detected in tested GC patients.

**Conclusion:**

The current study indicates that founder *BRCA1* mutations reported in Polish breast/ovarian cancer patients do not contribute to increased GC risk. The nine tested recurrent *BRCA1* and *BRCA*2 mutations were not detected in GC patients which may suggests that they are rare in GC patients in the Polish population. Further analyses, including sequencing of entire sequences of *BRCA1* and *BRCA2* genes, are necessary to ultimately determine the role of these two genes in GC in Poland.

## Introduction

Despite progress in diagnostic modalities, gastric cancer (GC) is still a challenge for medicine. On a global scale more than 700,000 people die each year of GC [1]. *Helicobacter pylori* is a Class 1 carcinogen that is consistently considered a major risk factor associated with GC, but it is still not clear why most infected individuals never develop this malignancy. Therefore, a genetic predisposition has been postulated and approximately 5 % of the total GC burden is associated with hereditary germline mutations in genes causing a highly penetrant, autosomal dominant predisposition [2]. One of the few known mutations that predisposes to the development of diffuse gastric cancer in young patients is the *CDH1* gene mutation [3, 4]. However, a study has shown that the *CDH1* gene mutation is not present among Polish families that meet hereditary diffuse gastric cancer (HDGC) criteria [5]. Recently, it has been shown that founder mutations in *CHEK2* gene are associated with increased GC risk in Polish patients. However, the observed risk was low (OR = 1.6, p = 0.004) and concerned only 8.7 % of 658 patients with gastric cancer unmatched for age or family history [6]. The role of other genes, in particular tumor suppressor genes that help repair damaged DNA and ensure the stability of cellular genetic material, is not clear. Although germline mutations in the tumor suppressor *BRCA1* and *BRCA2* genes are associated with a high risk of breast and ovarian cancer, however, they have also been shown to correlate with other cancers, including gastric cancer. There are several studies which investigated the role of *BRCA1/2* gene in gastric cancer in different populations. In a single Israeli study, selected *BRCA2* mutations were found in 5.7 % of 35 GC patients [7]. Other reports are mainly indirect and family-based studies, in which a risk of gastric cancer was analyzed on the basis of the observed and expected number or incidence of GC in *BRCA1/2* mutation carriers or their relatives [8–13]. Friedenson [14] has summarized these reports, analyzing data from more than 30 epidemiological studies on the incidence of other than the breast or ovarian cancers in *BRCA1* and *BRCA2* mutation carriers and in large populations eligible for mutation testing. He estimated an elevated risk for stomach cancer with RR = 1.69 (1.21-2.38) as well as other cancers including the pancreas, colon and prostate cancer. The majority of published analyses on the correlation between *BRCA1* and *BRCA2* mutations and GC are indirect and based on the observation of GC incidence in families with detected mutation, but do not show the prevalence of *BRCA1/2* mutations in patients with diagnosed gastric cancer. The distribution of *BRCA1* and *BRCA2* mutations in European populations is varied and is characterized by the genetic homogeneity or heterogeneity of a particular population [15]. Górski et al. [16] in a study of 66 familial breast/ovarian cancer patients reported 35 families with detected mutations, including 29 (82.9 %) being three founder mutations of *BRCA1* (C61G, 4153delA, 5382insC). Other studies confirmed statistically significant high contribution of three founder *BRCA1* mutations in Polish breast/ovarian cancer patients [17–21]. The contribution of these mutations was also investigated in other cancers. Cybulski et al. [22] analyzed *BRCA1* mutations (C61G, 4153delA, 5382insC) in a large group of 1793 patients with prostate cancer and 4570 population controls and found an association of C61G, 4153delA but not 5382insC with an increased prostate cancer risk (OR = 3.6, p = 0.045). In another Polish study, three *BRCA1* founder mutations were analyzed in a group of 2,398 colorectal cancer patients, but they were not associated with the overall cancer risk [23]. However, they were found to correlate with family history and younger age at diagnosis. Apart from founder mutations in Polish cancer patients, several other recurrent *BRCA1* (185delAG, 3819del5, 3875del4, 5370C > T) and *BRCA2* (886delGT, 4075delGT, 5467insT, 6174delT, 8138del5) mutations were detected [17–21, 24].

As a result of the demonstrated contribution of founder *BRCA1* mutations in Polish breast/ovarian and other cancers, it seems reasonable to investigate their role in gastric cancer cases. The purpose of this study was to determine the prevalence of three founder *BRCA1* mutations in unselected GC patients. In addition, the authors analyzed nine recurrent *BRCA1/BRCA2* mutations in GC cases.

## Patients and methods

### Patients

Three hundred and seventeen patients diagnosed between January 1999 and December 2007 at the Department of Gastroenterology, Pomeranian Medical University, Szczecin, Poland, with histopathologically confirmed GC, were included in this retrospective analysis. The baseline characteristics of the GC patients included in the study are shown in (Table 1). The mean age of all 317 cases (193 men and 124 women) was 59.5 ± 12.8 years (range 23–89 years). Of the 292 patients with a known family history, 156 (53.4 %) had cancer in first or second degree relatives and, of those, 32 (20.5 %) had breast or ovarian cancer, 60 (38.5 %) had gastric cancer in close relatives and 8 had both breast/ovarian cancer and gastric cancer in a close family member. Histological types were classified according to the Lauren classification [25]. Tumor location was classified as proximal (cardia and fundus) or other (body, pyloric part, entire stomach). Early GC was defined as invasive cancer that invades no more deeply than the submucosa, irrespective of lymph node metastasis.

**Table 1:** The clinicopathological features of GC patients

Characteristic	Total (n)	n (%)
Gender		
Male	193	60.9
Female	124	39.1
Total^a^	317	
Age^a^ (yr)	59.5 ± 12.8	
FHC		
No	136	42.9
Yes	156	49.2
Missing	25	7.9
Total	317	
Location^b^		
Proximal	70	22.1
Other location	247	77.9
Total	317	
Histology		
Intestinal	142	44.8
Diffuse	106	33.4
Mixed	45	14.2
Missing	24	7.6
Total	317	
Stage		
Early	48	15.1
Advanced	223	70.4
Missing	46	14.5
Total	317	

^a^Data are expressed as mean ± SD ^b^Tumor location

DS duration of symptoms FHC family history of cancer

### Controls

A control group for all GC cancer cases was previously analyzed and consisted of 4570 individuals, including 2000 newborn children from nine hospitals throughout Poland, 1570 adults selected from family doctors and 1000 individuals from Szczecin who submitted blood for paternity testing [22, 23].

A control group for GC male cases consisted of 3956 cancer-free men previously analyzed including 603 unselected men from family practice located in Szczecin, 1008 men were part of a population–based study to identify familial cancer, 1301 men at the age above 45 years and PSA (prostate-specific antigen) level below 4.0 μg/L selected randomly from a database of a population-based study and 1044 men who participated in a population colonoscopy screening program [26].

### Genotyping

Each patient involved it the study was informed about the aim and methods of the study and provided a written informed consent statement that was reviewed by our Institutional Ethics Committee. DNA from each patient was isolated from peripheral blood leucocytes. *BRCA1* and *BRCA2* mutation analysis was performed at the International Hereditary Cancer Center, Department of Genetics and Pathology, Pomeranian Medical University. All 317 patients were genotyped for the presence of three founder mutations in *BRCA1* (4153delA(c.4035delA); 5382insC (c.5266dupC); C61G(c.181 T > G) and additionally nine recurrent: fourth in *BRCA1* (185delAG (c.68_69delAG); 3819del5 (c.3700_3704del5); 3875del4 (c.3756_3759delGTCT); 5370C > T (c.5251C > T)) and five in BRCA2 (886delGT(c.658_659delGT); 4075delGT(c.3847_3848delGT); 5467insT(c.5239_5240insT); 6174delT(c.5946delT); 8138del5(c.7913_7917del5)) as previously reported in Polish women with breast and/or ovarian cancer [17–21, 24]. Genotyping of three founder *BRCA1* mutations was performed using allele-specific oligonucleotide polymerase chain reaction (ASA-PCR) (for 4153delA and 5382insC) and restriction fragment length polymorphism (PCR-RFLP) (for C16G), as previously described [16]. The nine recurrent *BRCA1/BRCA2* (185delAG, 3819del5, 3875del4, 5370C > T, 886delGT, 4075delGT, 5467insT, 6174delT, 8138del5) mutations were genotyped using TaqMan real-time PCR (Applied Biosystem/Life Technologies, Carlsbad, CA on Roche LightCycler 480). For the analysis of each mutation, the authors used positive, negative as well as non-template controls. The frequency of *BRCA1* founder mutations detected in GC patients was compared to a control group. Odds ratios (ORs) and 95 % confidence intervals (CIs) were calculated from 2-by-2 tables.

## Results

All patients were genotyped for the 3 founder and additionally 9 recurrent mutations. Out of all the tested *BRCA1/BRCA2* mutations only one, founder *BRCA1* (5382insC), was detected in two (0.63 %) of all 317 patients and in 1 % of a male group. Both cases of *BRCA1* mutation carriers were men (56 and 47 years old) and they both had intestinal, advanced gastric cancer located in body. In one case, there was a negative family history of any cancer; in the second case, the family history of cancers was unknown.

A *BRCA1* three founder mutation (C61G, 4153delA, 5382insC) was detected in 22 of 4570 (0.48 %) controls [22, 23]. No statistically significant differences between both cases and the control group were shown when comparing GC patients to controls (0.63 % vs. 0.48 %, OR = 1.3; 95 % CI 0.3-5.6, *p* = 0.71). When comparing 2 GC cases carrying *BRCA1* (5382insC) mutation with 17 of 4570 individuals carrying *BRCA1*(5382insC) mutation in the control population, there were no statistically significant differences between both groups (0.63 % vs. 0.37 %, OR = 1.7; 95 % CI 0.4-7.4, *p* = 0.48). Since both cases carrying *BRCA1* mutation involved male subjects, the authors compared a group of GC males to 3956 male controls described previously carrying 17 three founder (C61G, 4153delA, 5382insC) mutations including 13 *BRCA1* (5382insC) mutations [26]. A comparison of the two groups: cases and controls did not show statistically significant differences between both groups (1.0 % vs. 0.43 %, OR = 1.5; 95 % CI 0.3-6.4, *p = 0.61*). Similarly, when comparing GC male cases and male controls carrying only *BRCA1* (5382insC) mutation there were no statistically differences between both groups (1.0 % vs. 0.33 %, OR = 1.9; 95 % CI 0.4-8.6, *p = 0.39*).

## Discussion

Our data shows that 3 tested founder *BRCA1* mutations are not associated with gastric cancer. The result of the comparison of GC patients with controls is not statistically significant (OR = 1.3; 95 % CI 0.3-5.6, *p* = 0.71). The *BRCA1* (5382insC) was detected in two (0.63 %) of 317 GC patients. When comparing the prevalence of the BRCA1 (5382insC) in GC cases and controls, the results were still not statistically significant (OR = 1.7; 95 % CI 0.4-7.4, *p = 0.48*).

It should be underlined that only one *BRCA1* (5382insC) mutation was found in the gastric cancer group. In contrast to the current data, Cybulski et al. [22], who tested the same 3 founder mutations in *BRCA1*, concluded that *BRCA1* (5382insC) mutation is unlikely to be pathogenic for prostate cancer, but the increased risk was associated with the other two founder *BRCA1* mutations (4153delA and C61G). Similarly, Suchy et al. [23] made an attempt to establish the role of the same *BRCA1* (C61G, 4153delA, 5382insC) founder mutations in colorectal cancer. Interestingly, the obtained data did not show an increased risk of colorectal cancer in the carriers of the analyzed mutations (OR = 0.8; *p* = 0.8).

In the current study, both the *BRCA1* mutation carriers (1 %) were males out of 193 male cases. Speculating that perhaps an increased risk is gender-related, the group of GC male patients was compared with male controls. The obtained data did not confirm such a relationship (OR = 1.5; 95 % CI 0.3-6.4, *p = 0.61*). The men with BRCA1 mutations were not at an increased risk of gastric cancer. Similarly, the lack of carriers of the nine BRCA1 and BRCA2 recurrent mutation in tested GC cases was surprising in the light of the fact that there are several studies, which indicated that *BRCA1*/ *BRCA2* mutation carriers to be at an increased risk of GC and which are based on indirect research involving mainly breast/ovarian family-based studies. The Breast Cancer Linkage Consortium [8] reported an increased risk of GC in *BRCA2* families (RR = 2.59; 95 % CI 1.46–4.61) that was greater in carriers < 65 years old than in older carriers. Moran et al. [13] reported a significantly increased risk (RR = 2.4; 95 % CI 1.2–4.3) of *BRCA1* carriers developing GC cancer in a sample of 268 *BRCA1* and 222 *BRCA2* families. They also found a statistically significant risk of GC in *BRCA2* mutation carriers (RR = 2.7, 95 % CI 1.3–4.8) [13]. Based on data available for families eligible for *BRCA1*/*BRCA2* testing in the Swedish cancer registry, Bermejo and Hemminiki [12] found that GC before the age of 70 was twice as frequent (RR 2.04; 95 % CI 1.14–3.12) in families with breast and ovarian cancer than in the general population. Among many publications on that subject there are also those that assessed the risk as being much higher. Brose et al. [11], in a clinic-based study analyzing 483 *BRCA1* mutation carriers in 147 families, estimated the cumulative age-adjusted lifetime risk of GC as more than 5 (5.5 %, 95 % CI 3.4 to 7.5). Canadian authors compared 694 incident cases of ovarian cancer with the most commonly reported *BRCA1* or *BRCA2* mutations identified in 60 patients (between them the same as in our current study: 185delAG, 4075delGT, 6174delT) to 4378 relatives of cases who did not carry a mutation and estimated that the relative risk of GC in carriers of those mutations or relatives of cases was comparable to the previously cited author (RR = 6.2; 95 % CI 2–19) [10]. The same author analyzed 1171 unmatched ovarian cancer cases and investigated the presence of selected *BRCA1* and *BRCA2* mutations and, on the basis of his data, showed that the relative risk of developing stomach cancer in BRCA1 mutation carriers was 4.8 (95 % CI 1.5–15) [27].

The correlation between *BRCA2* gene mutations and gastric cancer was investigated in direct mutational analysis in an Israeli group. Figer et al. [7] found two (5.7 %) 6174delT *BRCA2* mutation carriers among 35 GC cases. They estimated that in Jewish individuals germline mutations in *BRCA2* seem to contribute to the genetic susceptibility toward gastric cancer. In our study, the authors did not detect BRCA2 gene mutations in GC patients. However, in considering these results it should be taken into account that: 1) the Jewish Ashkenazi population has a higher prevalence of harmful *BRCA1* and *BRCA2* mutations when compared to the global population; 2) only 35 GC cases were analyzed.

Similar data on the possible association between GC and *BRCA1* and *BRCA2* mutations were collected in Poland. In our previous study, of a group of 34 women with ovarian cancer and a family history of GC and 75 women with ovarian cancer and a family history of ovarian cancer but not stomach cancer were compared according to the presence of *BRCA2* mutations [28]. We found that almost 24 % of cases whose family members had stomach cancer carried a *BRCA2* mutation vs. 4 % without stomach cancer in the family history [28]. The results of this study corroborated the concept that stomach cancer is part of the spectrum of diseases associated with *BRCA2* mutations. In another report, Jakubowska et al. [29] identified *BRCA2* gene mutations (three frameshifts and three sequence variants) in 29 Polish families with an aggregation of at least one female breast and one male stomach cancer and observed significant differences between the incidence of *BRCA2* mutations in the breast/male stomach cancer families when this group was compared to the breast/ovarian cancer families (*p* < 0.025). This data supported a previous report that *BRCA2* is likely to be the molecular basis of stomach cancer. Despite the assessed risk of gastric cancer in *BRCA1/2* mutation carriers in families with an aggregation of breast/ovarian and gastric cancer in the current study, none of our 32 GC cases with a positive family history in first or second degree relatives of ovarian or breast cancer were *BRCA1* or *BRCA2* mutation carriers. Furthermore, of four women who had breast or ovarian cancer instead of GC, none were mutation carriers, which is incompatible with the Polish data on breast and ovarian cancer.

Taking into account the extensive body of literature evaluating the increased risk of developing GC in *BRCA1*/*BRCA2* mutation carriers, it seems that such a relationship is indisputable. However, there are reports that do not support this relationship. Thompson et al. [30] analyzed a cohort of 11,847 individuals from 699 families segregating a *BRCA1* mutation and could not make a compelling case for a significantly increased risk of GC associated with *BRCA1* gene mutation which is consistent with the results of our study.

## Conclusion

In conclusion, current data on the Polish population lead to the conclusion that *BRCA1* founder mutations do not contribute to increased risk of gastric cancer. The nine tested recurrent *BRCA1* and *BRCA2* mutations were not detected in GC patients which may suggests that they are rare in GC patients in the Polish population. Further research, including sequencing of entire sequences of *BRCA1* and *BRCA2* genes, is necessary to ultimately determine their role of these two genes in gastric cancer in Poland.

## Abbreviations

BRCA1: breast cancer susceptibility gene 1
BRCA2: breast cancer susceptibility gene 2
CDH1: cadherin 1 gene
